# Optimizing germination: comparative assessment of various growth media on dragon fruit germination and early growth

**DOI:** 10.1186/s12870-024-05247-6

**Published:** 2024-06-12

**Authors:** Ghulam Sarwar, Tauseef Anwar, Huma Qureshi, Muhammad Younus, Muhammad Waqar Hassan, Muhammad Sajid-ur-Rehman, Faizan Khalid, Faiza, Wajid Zaman, Walid Soufan

**Affiliations:** 1 Department of Botany, The Islamia University of Bahawalpur, Bahawalpur, Pakistan; 2 Department of Botany, University of Chakwal, Chakwal, Pakistan; 3 Department of Pharmacognosy, The Islamia University of Bahawalpur, Bahawalpur, Pakistan; 4 Department of Entomology, The Islamia University of Bahawalpur, Bahawalpur, Pakistan; 5 Department of Life Sciences, Yeungnam University, Gyeongsan, 38541 Republic of Korea; 6 Plant Production Department, College of Food and Agriculture Sciences King, Saud University, Riyadh, 11451 Saudi Arabia

**Keywords:** Growth media, Germination media, Cocopeat, Sand, Vermiculite, Peatmoss, Compost

## Abstract

Dragon fruit (*Selenicereus undatus*), known for its captivating appearance and remarkable nutritional profile, has garnered considerable attention in recent years. Despite its popularity, there's a dearth of research on optimal conditions for seed germination and early growth stages such as seedling shoot length, which are crucial for optimal crop yield. This study aims to bridge this gap by evaluating various growing media's performance on dragon fruit germination and early growth stages. Dragon fruit seeds were obtained from local markets in Pakistan and evaluated in five different growing media: cocopeat, peat moss, sand, vermiculite, and compost. Germination parameters were observed for 45 days, including seed germination percentage, mean germination time, and mean daily germination percentage, among others while early growth was monitored for 240 days. Statistical analysis was conducted using ANOVA and Tukey’s HSD test. Significant differences were found among the growing media regarding germination percentage, mean germination time, and mean daily germination. Vermiculite exhibited the highest germination rate (93.33%), while compost showed the least (70%). Peat moss and sand media facilitated rapid germination, while compost showed slower rates. Stem length was significantly influenced by the growth media, with compost supporting the longest stems. Vermiculite emerged as the most effective medium for dragon fruit seed germination, while compost showed slower but steady growth. These findings provide valuable insights for optimizing dragon fruit cultivation, aiding commercial growers and enthusiasts in achieving higher yields and quality. Further research could explore additional factors influencing dragon fruit growth and development.

## Introduction

Dragon fruit (*Selenicereus undatus)* is a fascinating tropical fruit that has garnered significant attention recently due to its unique appearance, delightful flavors, and remarkable nutritional profile. Belonging to the *Cactaceae* family, this exotic fruit is native to the Americas but has found widespread cultivation in various tropical and subtropical regions around the world [1]. Botanically, it is a perennial climber that grows quickly and has fleshy stems with a triangular shape, segmented stems with aerial roots that cling to surfaces, and rarely four or five-ridged stems. The plant produces stunningly vibrant fruits that range in color from brilliant shades of red to alluring shades of yellow or white, depending on the variety [2]. These fruits possess a distinctive, oblong shape with protruding scales or bracts, resembling the scales of a mythical dragon, hence the name “dragon fruit” [3, 4]. The alluring beauty of the flower of the pitaya plant is so captivating that it has earned an affectionate name like “Novel Woman” and “Queen of the Night” [5].

Besides beauty, dragon fruit contains high nutritional value and bioactive compounds, including powerful natural antioxidants [3]. Several reports suggest the therapeutic potential of dragon fruit against type 2 diabetes [6]. Dragon fruit contains high vitamin “C” content, which plays a crucial role in improving the immune system and promoting overall health. Additionally, dragon fruit is an excellent source of fiber, improving gut-related issues and avoiding constipation [7]. Dragon fruit also contains betalains and carotenoids that besides giving dragon fruit its vibrant color also possess potent antioxidant properties. These compounds help neutralize harmful free radicals in the body, thereby reducing the risk of chronic diseases and promoting overall cellular health [8]. Furthermore, dragon fruit is a good source of essential minerals like iron, magnesium, and potassium, which are vital for various bodily functions, including muscle contraction, nerve transmission, and blood pressure regulation [9].

Despite being nutritionally invaluable, there is a lack of comprehensive research investigating the optimal conditions for its seed germination and early growth stages. Dragon fruit breeders introduce new cultivars through conventional breeding and selecting the seedlings through grafting on mature root stalks for time-saving and early fruiting. This research is particularly relevant for the arid Bahawalpur district of Punjab, Pakistan, where optimizing dragon fruit seed germination can aid in establishing this drought-tolerant crop as an alternative for agricultural diversification and economic development in the region. Successful germination and establishment of seedlings are crucial steps in ensuring a robust and productive dragon fruit crop. Selecting the most suitable growing medium can significantly impact seed germination rates, seedling vigor, and overall plant health, ultimately affecting crop yield and quality [10]. Furthermore, identifying the medium that promotes optimal germination and seedling development can provide valuable insights for commercial growers, nursery operators, and hobbyists. Therefore, this study aimed to bridge this knowledge gap by systematically evaluating the performance of various growing media, including cocopeat, peat moss, sand, vermiculite, and compost on the germination and early growth stages of dragon fruit.

## Material and methods

### Plant material and study area

Fully ripened dragon fruits (for seed extraction) were bought from a local fruit market in Karachi, Pakistan. The variety of dragon fruit was confirmed to be *Selenicereus undatus* (Haw.) D.R.Hunt by Dr. Ghulam Sarwar of the Department of Botany, the Islamia University of Bahawalpur, Pakistan. The experimental site was a part of the study area of the Department of Botany, The Islamia University of Bahawalpur, Bahawalpur, Pakistan. The study area lies in the Cholistan desert located in Bahawalpur, Pakistan. It is located in the southwest of Punjab province (Pakistan). The total land area of the Cholistan desert is about 2.6 million hectares and has a length of about 480 km and width differs from 32 to 192 km. It is located at an elevation of 89 m above sea level. The average temperature and humidity during the study period was 28 °C and 60% respectively.

### Climatic conditions in the study area

Temperatures are high in summer and mild in winter, with no frost. In summer, temperatures may reach more than 51℃, and in winter they drop down below the freezing point. May and June are the hottest months, with a mean temperature of 34℃. Rainfall is a single source of fresh water in deserts. Average annual rainfall varies from 100 to 200 mm. It has been investigated that there are about 350 million cubic meters of runoff potential available for storage in the desert.

### Growth media

Five types of growth/germination media were used in the study namely cocopeat, peat moss, sand, vermiculite, and compost. A set of three seedling grow trays with 100 holes was used to evaluate the germination and growth of dragon fruit seeds in five different growth media. The tray was divided into five equal sections, each for a specific growth medium. The first 20 holes were filled with cocopeat, the next 20 with peat moss, followed by 20 holes filled with sand, another 20 with vermiculite, and the remaining 20 holes were filled with compost. Germination media were purchased from the Ms. Siraj-ud-Din Nursery- Garden Center (Plot # 88, Nazir Street, Garden Block, Garden Town, Lahore, Pakistan 54,000). A well-matured, nutrient-rich compost “ORGANIC COMPOST®”, (www.agrinfobank.com.pk) was purchased from the local market (C: 18%, N: 1.2%, P: 0.6%, K: 0.8% and Mg: 0.2%) with 7.8 pH value. Gray sand was thoroughly washed with tap water and rinsed with distilled water thrice, before autoclaving.

### Seed sowing

**Fig. 1:**
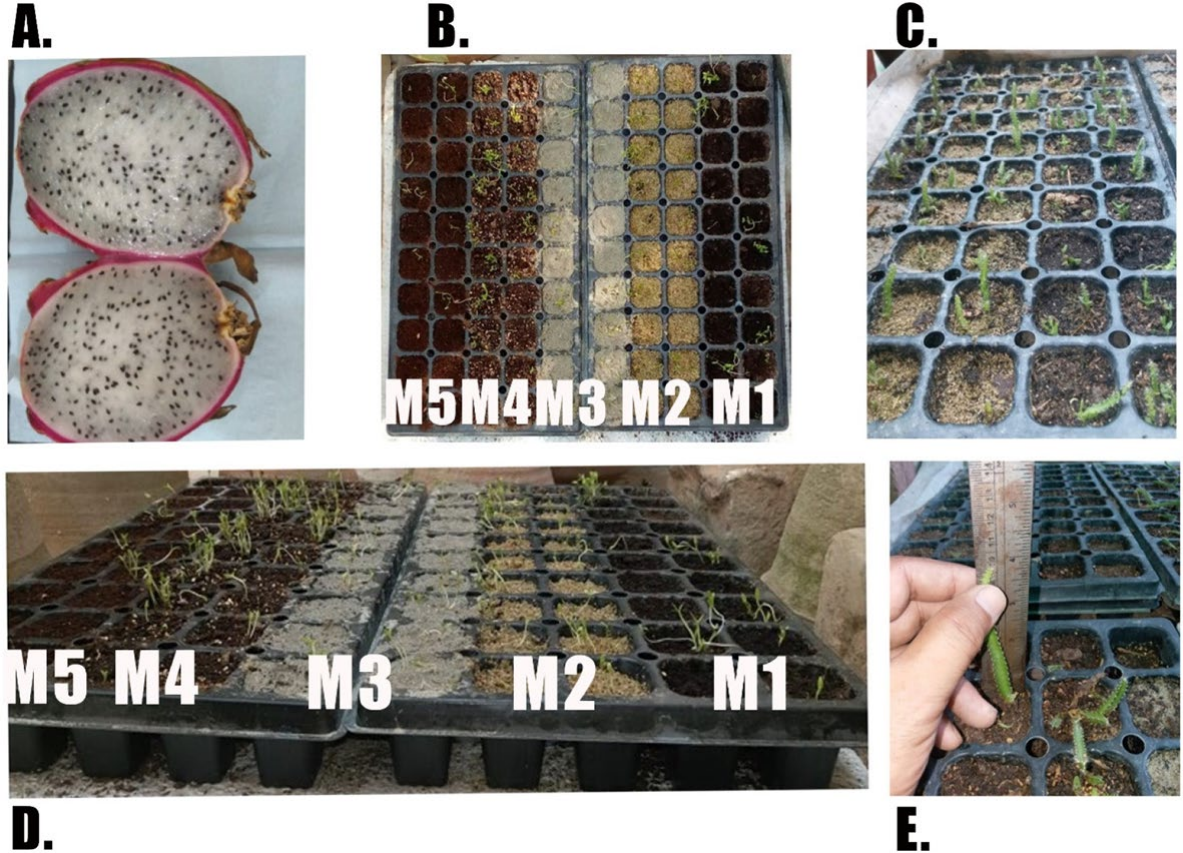
**A** Sliced dragon fruit for seed extraction. **B** Germination tray containing studied germination media; M1 being compost medium, M2 being vermiculite, M3 being sand, M4 being peat moss and M5 being cocopeat medium. **C** germinated dragon fruit plants with visible stems. **D** Dragon fruit plant seedlings at the initial stage of the experiment. **E** Measuring the stem length of dragon fruit plants across various growth media

The seeds of the dragon fruit plant were obtained from the fruit. The fruit was sliced horizontally into two halves. Using a fork or a spoon, the flesh was gently mashed to separate the seeds from the pulp. The seeds were then extracted and spread out on tissue paper placed on the plate, allowing them to dry and separate from any remaining pulp. Before sowing, the soil was moistened by adding water until it reached field capacity, ensuring optimal moisture levels for seed germination. Five seeds were then sown in each hole in the pre-moistened medium at a shallow depth. After that, the growing trays were placed in fully open sunny area to provide favorable conditions for germination. To maintain humidity and prevent excessive evaporation, the seedling tray was carefully covered with a cellophane sheet, to assist the germination process and protect it from birds (Fig. 1 A-E).

### Irrigation and maintenance

All the growing media were irrigated carefully with tap water using water shower bottle, providing only the required amount of water to prevent excessive moisture and fungal growth. To mitigate the risk of fungal infestations, a preventive measure was implemented. A 0.5% solution of copper sulfate fungicide was prepared by dissolving 2.5 g in 500 ml of water, and this solution was sprayed onto each hole of the seedling tray. This fungicide treatment was applied every 20 to 30 days to maintain a protective barrier against potential fungal pathogens.

### Seed germination parameters

Germination of seedlings was observed regularly in all the replications of each growth media for 45 days and later different germination-related parameters were calculated (Table 1).

**Table 1:** Different germination-related parameters studied and their formulas

Parameter	Formula	Description of Symbols	References
Germination Percentage (%)	$G\left(\%\right)\;=\;\frac{\sum_{i=1}^k\;n_i}N\;\times\;100$	ni = number of seeds germinated in the i^th^ time *N* = Total number of seeds used	[11]
Mean Germination Time (days)	$\overline t\;=\;\frac{\sum_{i=1}^k\;n_i}N\;\times\;100$	t̄ = Mean germination time; ni = number of seeds germinated at the ith time; ti = time (days) corresponding to the ith observation	[12]
Mean Germination Rate (day^−1^)	$\overline v\;=\frac1{\overline t}$	v̄ = Mean germination rate; t̄ = Mean germination time	[13]
Coefficient of Variation of Germination Time (CVt)	$CV_t=\frac{S_t}{\overline t}\;\times\;100$	CVt = Coefficient of variation of germination time; St = Standard deviation of germination time; t̄ = Mean germination time	[13]
Germination Index (GI)	$GI=\sum_{i=1}^kn_i/t_i$	GI = Germination index; ni = number of seeds germinated at ith time; ti = time (days) for germination at the ith time	[14]
Coefficient of Velocity of Germination (CVg)	$CVG=\frac{\sum_{i=1}^kn_it_i}{\sum_{i=1}^kn_i}\times\;100$	CVG = Coefficient of the velocity of germination; ni = number of seeds germinated at ith time; ti = time (days) corresponding to ith observation	[15]
Time to 50% Germination (days)	$T_{50}=\frac{t_i+\left({\displaystyle\frac{\sum_{i=1}^k\;n_i}2}-n_i\right)\left(t_j-t_i\right)}{n_j-n_i}$	T50 = Time to 50% germination; ni, nj = nearest cumulative number of seeds germinated; ti, tj = time (days) corresponding to ni and nj	[16]
Mean Daily Germination (MDG) Percent	$\overline G=\frac{GP}{T_n}$	G = Mean daily germination percent; GP = Final cumulative germination percentage; Tn = Total time intervals	[17]
Peak Value (PV)	Maximum quotient obtained by dividing successive cumulative germination values by the relevant incubation time	N/A	[17]
Germination Value	$GV=MDG\;\times\;PV$	GV = Germination value; MDG = Mean daily germination; PV = Peak value	[18]

### Seed germination percentage

The appearance of cotyledons was considered as germination (Iddrisu et al., 2019). The percentage of seed germination was calculated by following formulae mentioned in Table 1.

### Statistical analysis

The experiment was set up in a completely randomized design (CRD) with 20 replications for each treatment (cocopeat, peat moss, sand, vermiculite, and compost). Data was analyzed using Statistix 8.1 software. One-way ANOVA (analysis of variance) was computed to compare different treatments and later mean separation was performed with Tukey’s HSD test. For drawing plots, Origin (2024) software was used.

## Results and Discussion

### Germination percentage and relativized percentage

**Fig. 2:**
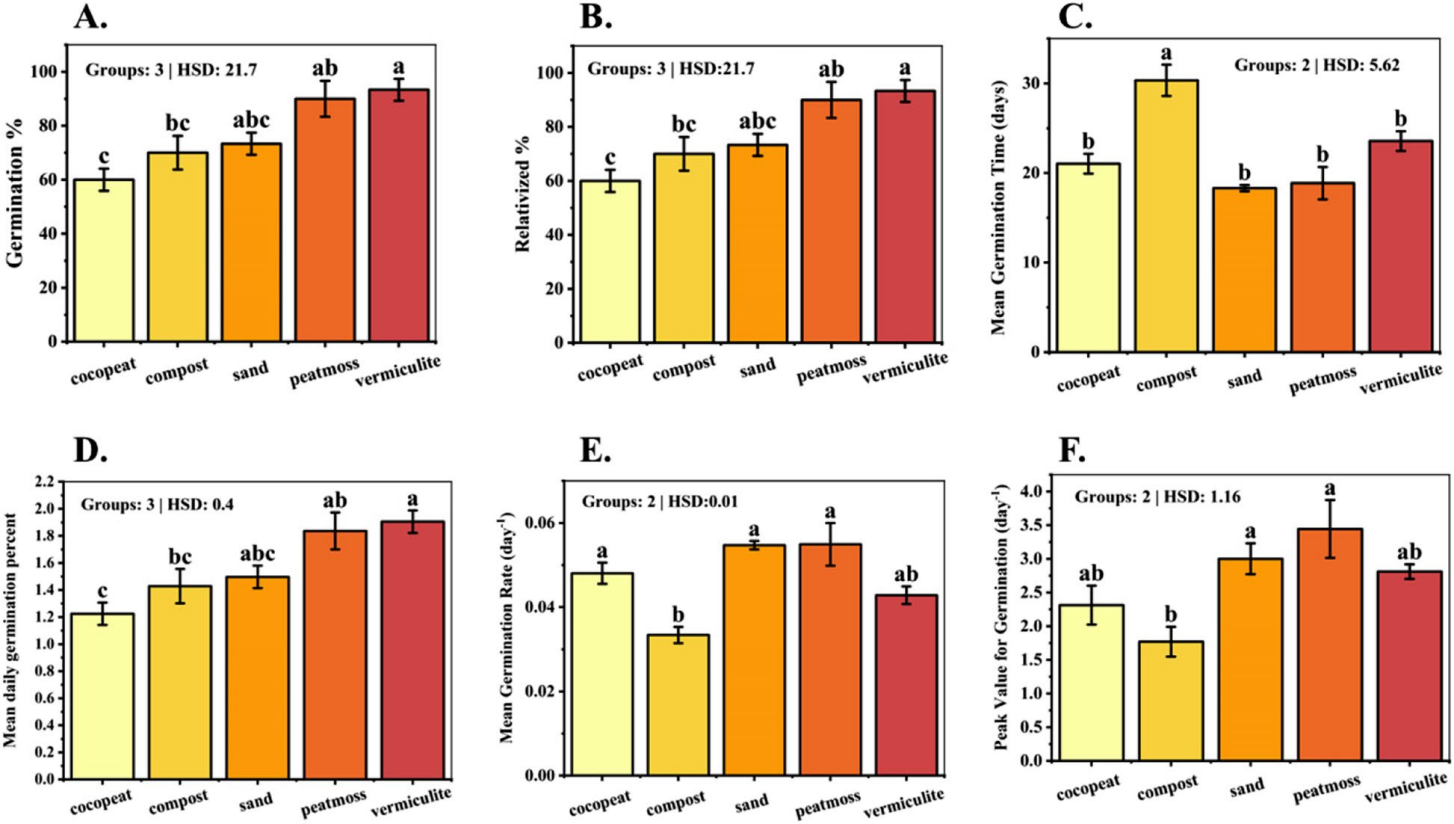
Bar plot of multiple comparisons of means using Tukey's Honest Significant Difference (HSD) test of (**A**) Germination %, **B** Relativized %, **C** Mean Germination Time, **D** Mean Daily Germination Percent, **E** Mean Germination Rate, **F** Peak Value for Germination. Means with different letters are significantly different at alpha 0.05 from each other

The analysis of variance (ANOVA) revealed statistically significant differences in the germination percentage (Fig. 2A) and relativized percentage (Fig. 2B) of dragon fruit seeds across the growth media (*p* < 0.001). The germination percentage of dragon fruit seeds varied considerably across the growth media evaluated. Vermiculite was the most effective medium, exhibiting the highest germination rate of 93.33%, followed closely by peat moss at 90%. The sand medium also performed reasonably well, with a germination percentage of 73.33%. Conversely, the cocopeat and compost media displayed comparatively lower germination rates of 60% and 70%, respectively. These findings suggest that the physical and chemical properties of vermiculite and peat moss, such as their water-holding capacity and aeration, were more conducive to the successful germination of dragon fruit seeds than the other media tested.

### Mean germination time

A statistically significant difference was found in the mean germination time (Fig. 2C) of dragon fruit seeds across all the growth media (*p* < 0.0001). The sand medium facilitated the earliest germination of dragon fruit seeds, with a mean germination time of 18.3 days statistically different from peat moss medium (18.87 days). In contrast, the compost medium exhibited the slowest germination, with seeds taking an average of 30.35 days to emerge. The vermiculite and cocopeat media occupied an intermediate position, with mean germination times of 23.56 and 21.03 days, respectively. The rapid germination observed in the sand and peat moss media can be attributed to their well-aerated and well-drained nature, providing favorable conditions for the germination process. In contrast, the compost medium exhibited the slowest germination, likely due to its higher water-holding capacity.

### Mean daily germination percentage

The evaluation of mean daily germination percentages across the growth media revealed distinct patterns in the germination dynamics of dragon fruit seeds (*p* < 0.001). Vermiculite emerged as the superior medium, exhibiting the highest mean daily germination percentage of 1.90%, closely followed by peat moss at 1.83% (Fig. 2D). This indicates that these two media facilitated a more rapid and consistent germination process, with a higher proportion of seeds germinating per day relative to the total number of germinated seeds. In contrast, the sand and compost media exhibited lower mean daily germination percentages of 1.49% and 1.42%, respectively. While the overall germination percentages for these media were reasonably high, the germination process appeared to be more gradual and protracted compared to vermiculite and peat moss.

### Mean germination rate (day^−1^)

The analysis of mean germination rates revealed notable differences among the growth media evaluated for dragon fruit seed germination (*p* = 0.0001). The peat moss and sand media exhibited the highest germination rates, with daily averages of 0.054 and 0.054, respectively (Fig. 2E). These were closely followed by the cocopeat and vermiculite media, which recorded mean germination rates of 0.048 and 0.04 per day, respectively. In contrast, the compost medium exhibited the slowest germination rate, averaging 0.03 per day. The observed variations in germination rates can be attributed to the unique physical and chemical characteristics of each growth medium, which influence factors such as water availability, aeration, and nutrient supply. The peat moss and sand media likely provided optimal conditions for rapid imbibition and subsequent metabolic activities essential for seed germination, resulting in higher daily germination rates.

### Peak value for germination

The analysis of peak germination values revealed significant differences among the five-growth media evaluated for dragon fruit seed germination (*p* = 0.0038). The peat moss medium exhibited the highest peak germination value of 3.44, indicating a rapid accumulation of germinated seeds early in the incubation period, followed by a tapering off in germination rate (Fig. 2F). This suggests that the conditions provided by peat moss were highly conducive to initiating and sustaining germination during the initial stages. The sand medium also exhibited a high peak germination value close to peat moss, suggesting a similarly rapid rate of early germination. The vermiculite and cocopeat media occupied an intermediate position, with peak germination values of 2.81 and 2.31, respectively, suggesting a more gradual accumulation of germinated seeds over time. In contrast, the compost medium exhibited the lowest peak germination value of 1.76, which may indicate suboptimal conditions for initiating and sustaining rapid germination, resulting in a more protracted and gradual germination process.

### Time taken for 10% germination (T10%)

**Fig. 3:**
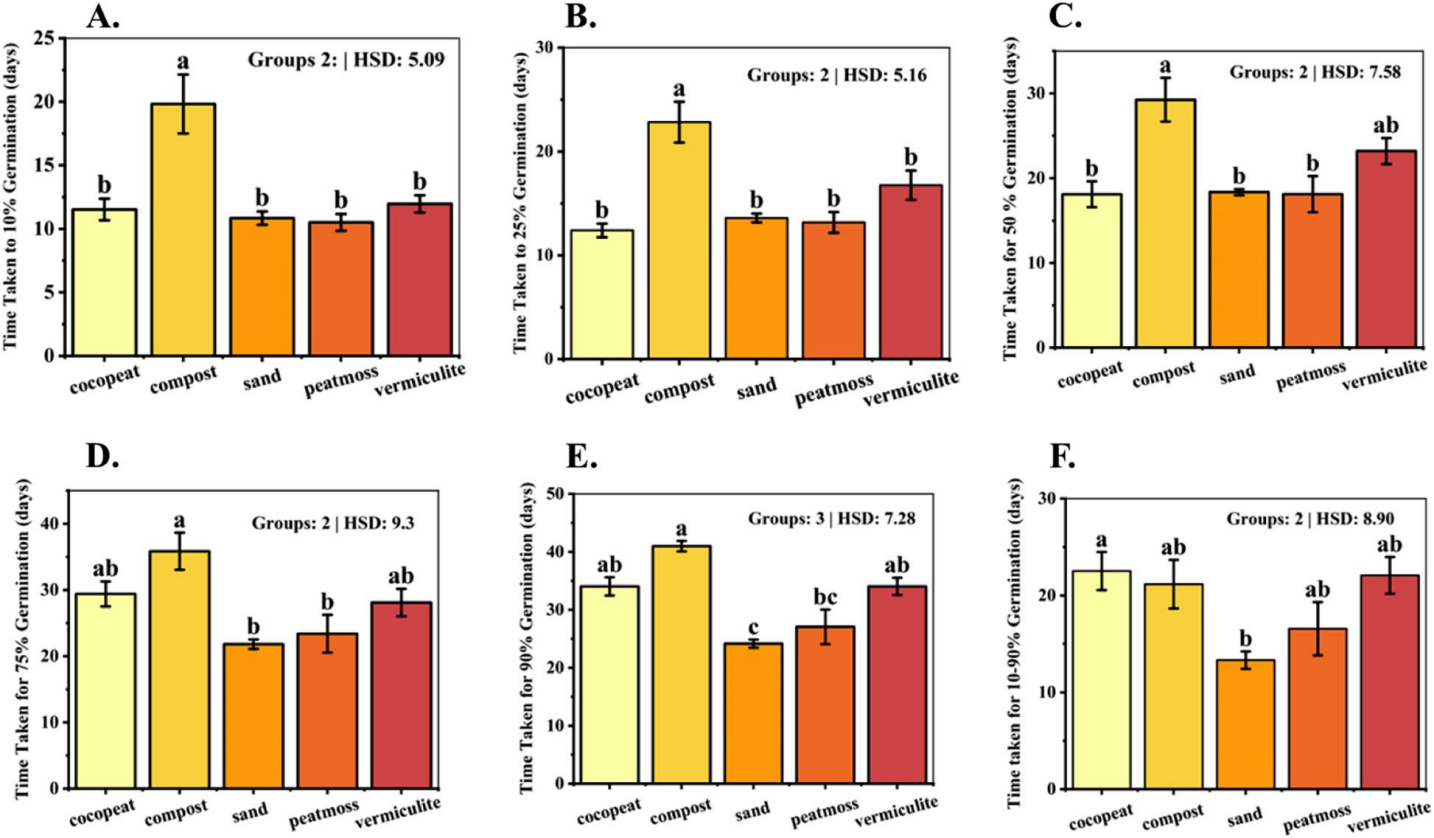
Bar plot of multiple comparisons of means using Tukey's Honest Significant Difference (HSD) test of (**A**) Time taken for 10% germination, **B** Time taken for 25% germination, **C** Time taken for 50% germination, **D** Time taken for 75% germination, **E** Time taken for 90% germination, **F** Time taken for 10–90% germination. Means with different letters are significantly different at alpha 0.05 from each other

The analysis of the time taken for 10% germination revealed notable variations among the growth media evaluated for dragon fruit seed germination (*p* = 0.0001). The compost medium exhibited the maximum time, with seeds reaching 10% germination in an average of 19.82 days. In contrast, the peat moss and sand media displayed an average of 10.5 and 10.84 days, respectively, required to achieve 10% germination. The vermiculite and cocopeat media exhibited an average time of 11.96 and 11.52 days, respectively, for achieving 10% germination (Fig. 3 A).

### T25%

Results revealed that the time taken for 25% germination differs significantly among the five-growth media (*p* < 0.001). The compost medium again exhibited the maximum time, reaching 25% germination in an average of 22.82 days (Fig. 3B). In contrast, the peat moss and sand media took an average of 13.17 and 13.6 days, respectively, to achieve 25% germination. The vermiculite medium took 16.75 days for 25% germination.

### T50%

The analysis of the time taken for 50% germination revealed significant differences among the five-growth media (*p* = 0.0008). Time taken for 50% germination showed a similar trend as exhibited by T10% and T25%. The compost medium exhibited a maximum time of 29.25 days (Fig. 3C). In contrast, the peat moss, sand, and cocopeat media displayed an average of 18.11, 18.35, and 18.1 days, respectively, to achieve 50% germination. The vermiculite medium took an average of 23.2 days required for 50% germination. These variations can be attributed to the unique physical and chemical characteristics of each growth medium, influencing factors such as water availability, aeration, and nutrient supply, which regulate the progression of seed germination.

### T75%

Time taken for 75% germination differs significantly among studied growth media (*p* = 0.0019). The sand medium exhibited the fastest germination rate, reaching 75% germination in an average of 21.825 days, outperforming the other media (Fig. 3D). The peat moss medium also displayed a relatively rapid germination rate, with an average of 23.4 days required for 75% germination. In contrast, the cocopeat, vermiculite, and compost media exhibited slower germination kinetics, requiring an average of 29.4, 28.1, and 35.85 days, respectively, to achieve 75% germination.

### T90%

The analysis of the time taken for 90% germination also revealed significant differences across the studied growth media (*p* < 0.001). Time taken for 90% germination showed quite a similar trend as exhibited by T75%. The sand medium exhibited the fastest germination rate with an average of 24.16 days (Fig. 3E). The peat moss medium also displayed a relatively rapid germination rate, with an average of 27.06 days. In contrast, the cocopeat, vermiculite, and compost media exhibited slower germination kinetics, requiring an average of 34.04, 34.04, and 40.98 days, respectively, to achieve 90% germination. These findings suggest the potential of sand to be used as an efficient medium for attaining quick germination.

### T10-90%

The analysis of the time taken for 10% to 90% germination revealed notable variations across all the studied growth media (*p* = 0.0217). The sand medium exhibited the fastest germination rate, taking an average of 13.32 days for this range (Fig. 3F). The peat moss medium also displayed a relatively rapid germination rate, with an average of 16.56 days required for the 10% to 90% germination range. In contrast, the cocopeat, vermiculite, and compost media exhibited slower germination kinetics, requiring an average of 22.52, 22.08, and 21.16 days, respectively, for the same range.

### Coefficient of variation of germination time (CV_t_)

**Fig. 4:**
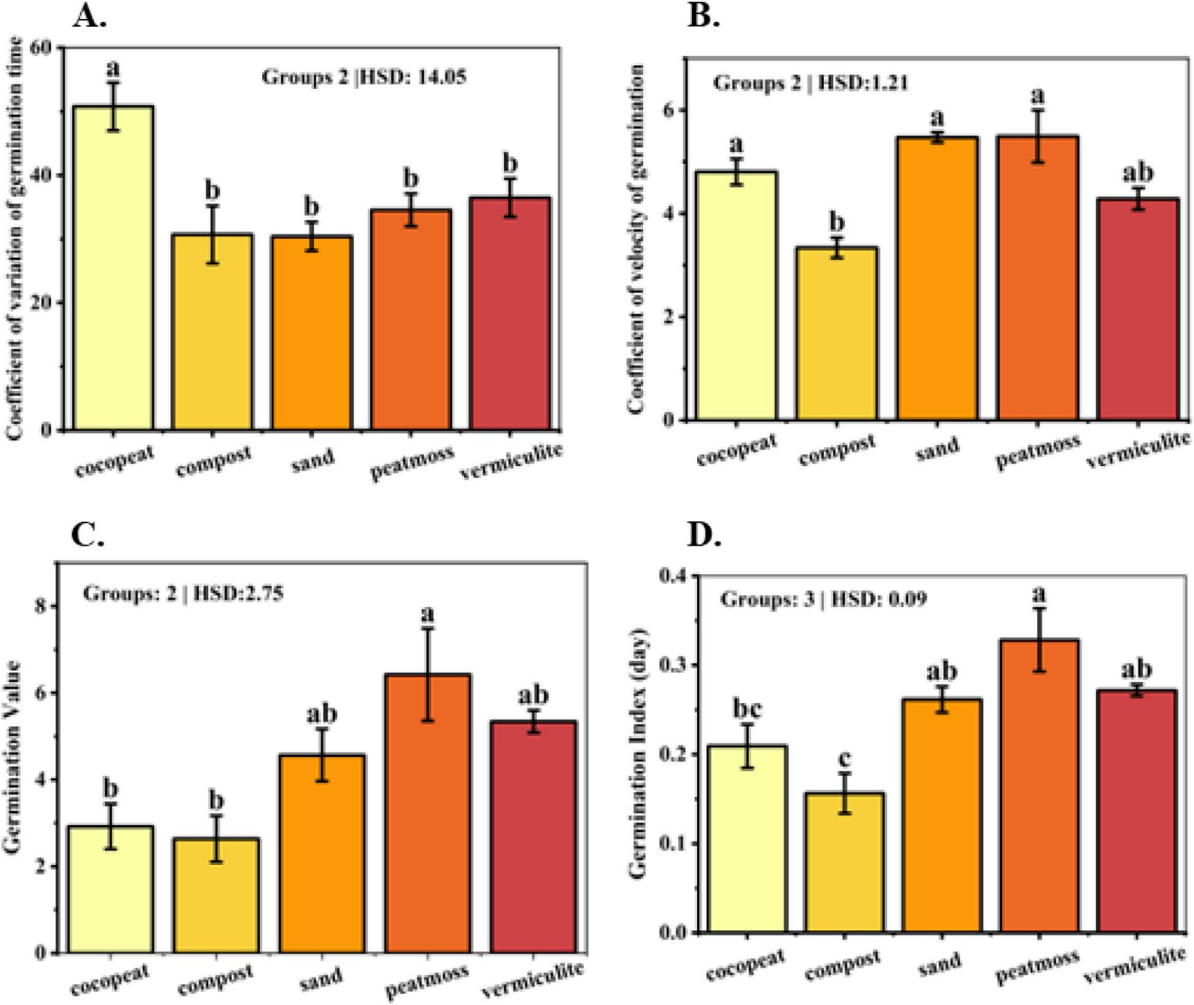
Bar plot of multiple *comparisons* of means using Tukey's Honest Significant Difference (HSD) test of (**A**) coefficient of variation of germination time, **B** coefficient of velocity of germination, **C** Germination value, **D** Germination Index. Means with different letters are significantly different at alpha 0.05 from each other

CVt is a measure used to quantify the relative variability of germination times within a sample of seeds. ANOVA revealed a significant difference in CVt across the five media (*p* = 0.0019). The cocopeat medium exhibited the highest coefficient of variation, with a mean value of 50.76239, indicating substantial variation in the germination times of individual seeds within this treatment (Fig. 4A).

In contrast, the compost and sand media displayed significantly lower coefficients of variation, with mean values of 30.6995 and 30.40608, respectively, suggesting more uniformity in germination times. The peat moss and vermiculite media occupied an intermediate position, with mean coefficients of variation of 34.56422 and 36.47845, respectively.

### Coefficient of velocity of germination (CVg)

The analysis of the CVg revealed notable variations across the studied growth media (*p* < 0.001). The peat moss and sand media exhibited the highest CVg, with mean values of 5.49257 and 5.4718, respectively, indicating rapid germination rates. The vermiculite and cocopeat media displayed intermediate coefficients, with mean values of 4.2836 and 4.80674, respectively. The compost medium exhibited the lowest coefficient of 3.33916, indicating a relatively slower germination rate compared to the other media (Fig. 4B).

### Germination value

The evaluation of germination values, which integrate both the speed and completeness of germination, revealed significant differences among the growth media used for dragon fruit seed germination (*p* = 0.0023). The peat moss medium exhibited the highest germination value of 6.42157, indicating a superior combination of rapid germination kinetics and a high overall germination percentage. This suggests that the physical and chemical characteristics of peat moss provided optimal conditions for efficient and successful seed germination. The vermiculite medium also demonstrated a relatively high germination value of 5.34331, indicating its effectiveness in facilitating both rapid and complete germination, albeit slightly lower than that observed in peat moss. Notably, the sand medium, despite exhibiting a lower germination value of 4.56676, still ranked among the top performers, suggesting its potential as an alternative growth medium for dragon fruit seed germination. In contrast, the cocopeat and compost media exhibited comparatively lower germination values of 2.92337 and 2.63643, respectively. While these media supported seed germination to a certain extent, their performance was inferior to the top-ranking media in terms of the combined factors of germination speed and completeness (Fig. 4C).

### Germination Index (GI)

The analysis of the GI revealed a significant difference (*p* = 0.0005) across the growth media. The peat moss medium exhibited the highest GI (mean 0.32822), indicating rapid and efficient germination (Fig. 4D). The vermiculite and sand media also displayed high germination indices, with mean values of 0.27186 and 0.26149, respectively. The cocopeat and compost media exhibited GI of 0.20923 and 0.15632, respectively, indicating slower and less efficient germination compared to the other media.

### Stem length

**Fig. 5:**
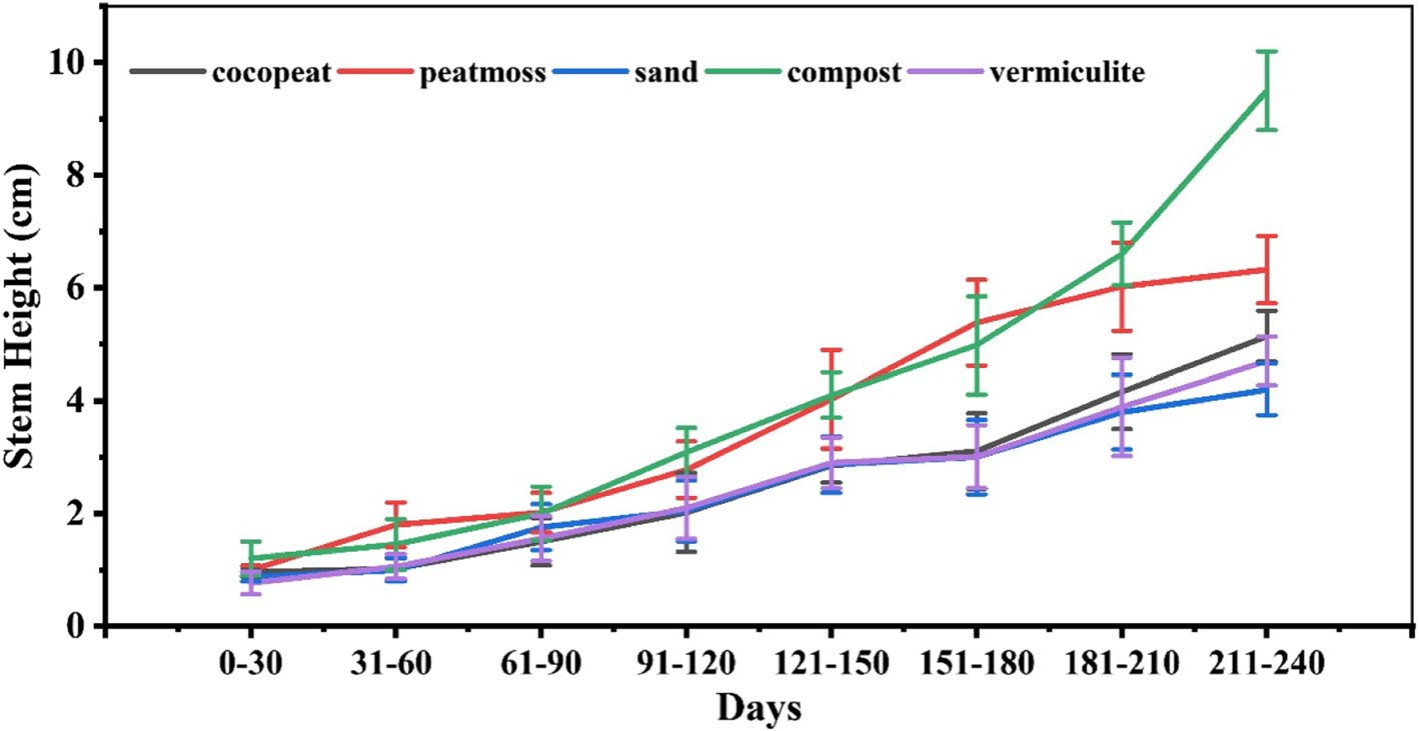
Bar plot of multiple comparisons of means using Tukey’s Honest Significant Difference (HSD) test of Stem length of Dragon fruit plant. Means with different letters are significantly different at alpha 0.05 from each other

The growth media had a significant impact on the stem length of dragon fruit seedlings (Fig. 5). In the initial stages (0–30 days), the stem length of the dragon fruit plant was comparable across all media, ranging from 0.77 ± 0.2 cm in vermiculite to 1.2 ± 0.3 cm in compost.

However, as time progressed, distinct differences emerged. Peat moss and compost consistently exhibited superior stem elongation, with mean lengths of 6.02 ± 0.78 cm and 6.6 ± 0.56 cm, respectively, between 181–210 days. Conversely, sand and cocopeat supported relatively slower growth, with mean stem lengths of 3.8 ± 0.66 cm and 4.16 ± 0.66 cm during the same period. Vermiculite demonstrated an intermediate performance, with a mean stem length of 3.89 ± 0.87 cm at 181–210 days. By the end of the observation period (211–240 days), compost outperformed all other media, yielding the maximum mean stem length of 9.5 ± 0.7 cm, followed by peat moss (6.32 ± 0.6 cm), vermiculite (4.7 ± 0.43 cm), cocopeat (5.14 ± 0.45 cm), and sand (4.2 ± 0.45 cm). This was anticipated due to better and constant nutrient supply provided by compost medium.

The current study investigated the role of different germination media namely cocopeat, peat moss, sand, vermiculite, and compost on the germination and early growth of dragon fruit plants. The process of germination holds significant importance in the growth of any species because the seedling establishment depends greatly on seed germination. The germination phase includes a sequence of events, beginning with imbibition and ending in the emergence of the radicle and plumule. The process of germination is greatly influenced by the interplay between environmental factors and the genetic constitution of the plant species [19].

An important environmental factor regarding the successful germination of seeds is the germination medium. The growing or germination medium exerts a direct influence on the development and subsequent maintenance of seedlings. Not all soils used for nursery operations and cultivation are inherently suitable for seed germination and subsequent seedling growth. Consequently, the judicious selection and utilization of appropriate growing media or substrates are crucial to producing high-quality plants [20]. An optimal growing medium ensures adequate anchorage or structural support for the plant, facilitating nutrient and water acquisition. Additionally, it enables efficient oxygen diffusion to the roots and permits gaseous exchange between the roots and the surrounding atmosphere beyond the root substrate [21]. It also plays a pivotal role in ensuring optimal seedling quality [22]. Generally, growing media can be categorized into two types such as soil-based and soilless (organic-based) media. Organic-based media comprises a blend of compost, peat, cocopeat, and other organic materials mixed with inorganic ingredients [23].

In the current study, vermiculite showed the maximum germination percentage (93.33%). Vermiculite has been extensively used for years to amend professional potting soils derived from peat moss [24]. Vermiculite is widely used for optimum germination as it facilitates aeration and drainage, and possesses substantial water retention. Our results align with [25] who reported that vermiculite shows maximum seed germination as compared to other growth mediums. High germination % in vermiculite medium is also in agreement with the results of [20] who have observed a maximum germination percentage of 92.7% in vermiculite medium. [26] has also documented similar results favoring vermiculite for maximum germination percentage. The minimum germination % was found in the cocopeat medium. However, our results are contrary to the number of studies that have presented cocopeat as the potential medium for optimal germination percentage. Dharmveer et al. [27] have documented that cocopeat in combination with sand and soil (1:1:1), greatly augmented the germination percentage in *Angelica glauca*. Similar findings have also been presented by [28] in *Syzygium cumunii.*

The stem length was found maximum in compost medium (6.6 ± 0.56 cm). Similar findings have also been presented by [20]. He has reported maximum shoot length in compost and sand media (1:1) in papaya. Dharmveer et al. [27] have also documented similar findings in *Angelica glauca.* The authors showed that compost along with sand, soil, and cocopeat in a 1:1:1:1 had the maximum positive effect on germination and seedling growth. Our findings are also in agreement with the findings of [29] who have documented compost to be the best medium for seedling growth. These positive effects on the seedling growth are anticipated to be due to nutrients provided by the compost.

## Conclusion

In conclusion, the study delves into the pivotal role of germination media in influencing the growth and development of dragon fruit (*Selenicereus undatus*) seedlings. The investigation underscores the significance of selecting appropriate growing media to ensure optimal germination rates, seedling vigor, and subsequent growth stages. Among the media evaluated, vermiculite emerged as the most effective medium, facilitating the highest germination percentage and demonstrating favorable characteristics such as aeration and water retention. Peat moss also exhibited promising results, closely following vermiculite in terms of germination performance. Conversely, cocopeat and compost displayed comparatively lower germination rates, highlighting the importance of understanding the unique properties of each medium. Furthermore, the study sheds light on the importance of stem length as an indicator of seedling growth. Compost emerged as the most conducive medium for promoting stem elongation, likely due to its nutrient-rich composition supporting sustained growth over time. By providing valuable insights into the performance of different media, this study contributes to optimizing cultivation practices and enhancing crop productivity. Additionally, the study underscores the need for further research to explore additional factors influencing dragon fruit germination and seedling growth, thereby advancing our understanding and facilitating sustainable production practices for this fascinating tropical fruit.

## Data Availability

The author confirms that all data generated or analyzed during this study are included in this published article.
